# An innovative program to provide methodological mentoring and to foster the development of robust research teams for K awardees: RAMP Mentors

**DOI:** 10.1017/cts.2020.536

**Published:** 2020-09-04

**Authors:** Laura J. Rasmussen-Torvik, Lauren A. Daniels, Keith A. Herzog, Emily J. Traw, Michael F. Fleming, Richard M. Pope, Donald M. Lloyd-Jones, Leah J. Welty

**Affiliations:** 1 Department of Preventive Medicine, Northwestern University Feinberg School of Medicine, Chicago, IL, USA; 2 Northwestern University Clinical and Translational Science Institute, Chicago, IL, USA; 3 Department of Psychiatry and Behavioral Sciences, Northwestern University, Chicago, IL, USA; 4 Department of Medicine, Northwestern University, Chicago, IL, USA

**Keywords:** Mentoring, research teams, research methods, education, career development awards

## Abstract

Within the Biostatistics, Epidemiology, and Research Design (BERD) component of the Northwestern University Clinical and Translational Sciences Institute, we created a mentoring program to complement training provided by the associated Multidisciplinary Career Development Program (KL2). Called Research design Analysis Methods Program (RAMP) Mentors, the program provides each KL2 scholar with individualized, hands-on mentoring in biostatistics, epidemiology, informatics, and related fields, with the goal of building multidisciplinary research teams. From 2015 to 2019, RAMP Mentors paired 8 KL2 scholars with 16 individually selected mentors. Mentors had funded/protected time to meet at least monthly with their scholar to provide advice and instruction on methods for ongoing research, including incorporating novel techniques. RAMP Mentors has been evaluated through focus groups and surveys. KL2 scholars reported high satisfaction with RAMP Mentors and confidence in their ability to establish and maintain methodologic collaborations. Compared with other Northwestern University K awardees, KL2 scholars reported higher confidence in obtaining research funding, including subsequent K or R awards, and selecting appropriate, up-to-date research methods. RAMP Mentors is a promising partnership between a BERD group and KL2 program, promoting methodologic education and building multidisciplinary research teams for junior investigators pursuing clinical and translational research.

## Introduction

In 2006, the National Institutes of Health established Clinical and Translational Science Awards (CTSAs) to “advance the assembly of institutional academic ‘homes’ that can provide integrated intellectual and physical resources for the conduct of original clinical and translational science” [1]. The overall goal of the CTSAs was “to ensure that extraordinary scientific advances of the past decade will be rapidly captured, translated, and disseminated for the benefit of all Americans” [1]. CTSAs include a Biostatistics, Epidemiology, and Research Design (BERD) component, to enhance “innovation, access, and quality” of research methods, with “continuing education elements to provide support to investigators for translational research design” [2,3]. Another critical, integrated aspect of the CTSAs has been training junior investigators to conduct clinical and translational research through the Multidisciplinary Career Development Program (KL2), which may be awarded in conjunction with a CTSA.

The CTSA-funded Northwestern University Clinical and Translational Sciences Institute (NUCATS) was established in 2008, along with the accompanying Northwestern Multidisciplinary Career Development KL2 scholars program. Junior clinical and translational investigators are appointed as KL2 scholars on a competitive basis and receive funding for their research as well as career development resources for 2–3 years. From 2008 through 2019, NUCATS has appointed 28 KL2 scholars. In 2015, the NUCATS BERD group partnered with the KL2 program to develop a new educational mentoring program for KL2 scholars, called Research design Analysis Methods Program (RAMP) Mentors.

Clinical and translational research involves larger and more complex data than ever before, and RAMP Mentors was designed to address a growing need for more integrated, consistent methodological support for KL2 scholars. KL2 awards by design provide limited salary support for primary mentors, and no salary support for other faculty mentors, such as biostatisticians. RAMP Mentors was therefore conceived to address increasing KL2 scholar needs in biostatistics, epidemiology, and broadly associated fields such as bioinformatics, health economics, computational biology, and qualitative research methods. An essential component of RAMP Mentors is providing salary support to mentors to enable full investment in their mentorship of KL2 scholars.

RAMP Mentors pairs each KL2 scholar with one or two mentors with relevant methodological expertise for individualized training and collaboration. The long-term objectives of RAMP Mentors are (1) to create robust research teams capable of carrying out complex, multidisciplinary clinical and translational research and (2) to improve the quality and sophistication of KL2 scholars’ work, leading to more publications and higher rates of successful competition for independent funding. In this article, we describe the implementation of RAMP Mentors through 2019, including KL2 scholar assessments of the program and surveys examining the confidence of Northwestern University K awardees (both KL2 and others) to establish methodologic collaborations. For simplicity, we refer to KL2 awardees as scholars, we refer to methodologic mentors appointed by RAMP as “mentors” unless otherwise noted, and we refer to primary, clinical mentors who are named in the KL2 application as “primary” mentors.

## Methods

### Design and Implementation of RAMP Mentors

RAMP Mentors has been offered to scholars appointed to the Northwestern University institutional KL2 training grant since 2015. Once scholars are appointed to the KL2 program, the RAMP Mentors director and co-director (an epidemiologist and a biostatistician) work to identify potential mentors. After reviewing the KL2 scholar’s application, they identify two or three areas in which the scholar would benefit from additional support. These are generally areas in which the scholar, primary mentor, and any other named mentors and/or collaborators on the KL2 application do not have significant prior training or expertise, such as cohort study design and implementation, analysis of whole genome sequence data, or mixed models regression analysis. Once these areas are identified, the directors create a list of potential mentors for each perceived methodologic need, then meet with the KL2 scholar and their primary mentor to discuss potential pairings. In some cases, KL2 scholars and their primary mentors might suggest additional areas of methodologic focus and possible mentors. The RAMP Mentors director, the KL2 scholar, and the KL2 primary mentor then reach consensus on one or two RAMP-appointed mentors to approach. Finally, the RAMP Mentors director approaches the potential mentors to determine their willingness to serve for at least 1 year.

Expectations for both mentors and KL2 scholars are explained in an in-person meeting before the scholar and mentor begin regular meetings. It is expected that scholars and mentors will meet at least monthly but may meet more often if they wish. The format and content of the meetings are determined by the mentor and scholar and may include a variety of activities, for example, they may be used for discussion of a journal article highlighting a relevant methodology or for conducting a guided tutorial of an analysis program, similar to activities one might pursue as part of an independent study. The meetings may also be used for discussions about study design, data analysis, manuscript preparation, or responding to reviewers. Meetings need not focus solely on the KL2 research project but may also cover other ongoing research or future research planning for subsequent K or R applications. Mentors are expected to provide methodologic *guidance* about data analysis but are not expected to perform analyses for the KL2 scholar. If the scholar has analytic support funded through their KL2 award or another mechanism, they are able to bring an analyst (e.g., master’s-level biostatistician, database manager) to the scholar/mentor meeting, but the analyst and mentor do not meet without the KL2 scholar. The KL2 scholar is welcome to bring additional people to scholar/mentor meetings, such as the primary mentor or a postdoctoral student, as long as the presence of the additional person does not infringe on the meeting addressing the methodologic needs of the scholar. This organizational structure ensures that the mentor’s time is fully dedicated to supporting the scholar’s research portfolio.

Mentors appointed by RAMP have a small percent effort dedicated to the program, typically 2.5% per scholar, supported by the BERD budget within the CTSA award. This protected time is meant to (1) reinforce the idea that the mentor must make time monthly to meet with the scholar, even if there are many other competing demands on his or her time and (2) allow the role of the KL2 mentor – and the connection between mentor and scholar – to be formally recognized in National Institutes of Health biosketches. Mentors and scholars are as necessary reminded that the RAMP Mentors program does not involve consulting or work for hire. When the mentor’s contributions meet the standards for authorship, scholars include mentors as co-authors on manuscripts [4].

### RAMP Focus Group Assessment

The Searle Center for Teaching and Learning at Northwestern University (https://www.northwestern.edu/searle/services/feedback-on-teaching/end-of-term-focus-group.html) conducts biennial focus groups with the KL2 scholars to solicit feedback about all aspects of the KL2 program, including RAMP Mentors. These focus groups are held every other year so that all scholars have the opportunity to participate one time during their KL2 appointment. Questions are generated by the KL2 Leadership team ahead of time and sent to the Searle Center for the facilitator to use, although the facilitator has the leeway to pursue other lines of questioning during the focus group. The Searle Center regularly conducts focus groups for Northwestern University educational programs; they are familiar with but external to NUCATS and are able to provide subjective reports and commentary. Reports generated from the transcripts of two focus groups, conducted in September 2016 and October 2018, were used for this article.

### RAMP Survey Assessment

To provide feedback on the entire KL2 program, scholars also complete web surveys (via REDCap [5]) at 1 year (mid-point) and 2 years (final) after their appointment. These surveys include open-ended and Likert scale questions assessing the RAMP Mentors program. Results from five scholars’ final evaluations and three scholars’ mid-point evaluations are included in this article. Mid-point evaluations were used only when final evaluations were not available for a scholar. A copy of the survey questions for the final (2- year) assessment is available in the supplement. In addition to questions specific to RAMP Mentors, the surveys included general questions on scholars’ confidence in: (1) seeking support and advice from colleagues with expertise in research methods; (2) asking questions and communicating with colleagues with expertise in research methods; (3) collaborating with colleagues with methodological expertise; (4) obtaining funding to support research activities; (5) securing subsequent K- or R-series awards; and (6) selecting appropriate, up-to-date, and novel research methods. For comparison and benchmarking, in April 2018 we distributed these six questions to other Northwestern University K awardees who had been appointed recently, within the previous 3 years (*n* = 68 total; 37 female, 31 male). Surveys were conducted in REDCap [5] and distributed via email. To encourage participation and honest feedback, we distributed the survey as a public link and did not track individual responses or ask for identifying or other demographic information. A copy of this survey is also available in the supplement. Institutional review board approval was not required because the data were collected anonymously for quality improvement efforts.

### Data Analysis

Survey responses were collected in REDCap [5] and summarized in R statistical software [6]. Likert responses for the six confidence questions distributed to both KL2 scholars and other Northwestern University K awardees were coded from 4 “very true” to 1 “not true at all.” Although our approach was exploratory, we used two-sample, two-sided *t* tests to compare average responses of NUCATS KL2 scholars with other Northwestern University K awardees on the six common survey questions. Likert responses are ordinal, but the *t* test has been shown to be robust to this departure from normality [7]. We report mean differences and associated 95% confidence intervals; intervals that do not contain 0 indicate statistical significance at the 0.05 level. We did not correct for multiple comparisons. We used StatTag [8] to connect statistical output to manuscript results.

## Results

### Mentor/Scholar Pairs

From inception through summer of 2019, RAMP Mentors have assigned 16 different methodologic mentors to 8 KL2 scholars (5 female; 3 male). Seven scholars have completed the KL2 program. One scholar is in the third year of their appointment. (In the fall of 2019, a new class of four KL2 scholars was matched with four RAMP-appointed mentors; because evaluation data are not yet available for this group, they are not included in this article.) Mentors are not prohibited from mentoring multiple scholars, although to date no mentor has been paired with more than one scholar through RAMP Mentors. This is largely because of the diversity of the methodologic needs of the KL2 scholars and the individualized selection of mentors. Table 1 summarizes the expertise of the KL2 scholars and their RAMP-appointed mentors. Three scholars have had a single methodologic mentor, three scholars have had two mentors, and two scholars have had three mentors (switching mentors after 1 year when their methodologic needs changed). Mentors included experts in biostatistics, epidemiology, and bioinformatics (next-generation sequencing analysis, RNA-Seq analysis, and image analysis). Methodologic mentoring in qualitative data analysis is also available but has not yet been needed. KL2 scholar/mentor pairs have to date published eight articles together (including four that each included two mentors). Of the seven scholars who have completed their KL2 awards, five have received subsequent NIH funding as principal investigator to date (two K08s; three K23s).

**Table 1.** KL2 scholar/RAMP-appointed mentor pairings

KL2 scholar	KL2 scholar field of study	Field of study for RAMP-appointed mentors
1	Neurology	1) Biomedical imaging 2) Epidemiology (cohort study design and analysis) 3) Biostatistics^*^
2	Gastroenterology and Hepatology	1) Biostatistics (methods for longitudinal data) 2) Epidemiology
3	Neurological Surgery (Pediatrics)	1) Bioinformatics (RNA-Seq) 2) Biostatistics
4	Allergy and Immunology	1) Epidemiology (study design and analysis) 2) Bioinformatics (RNA-Seq)
5	Neurological Surgery	1) Biostatistics 2) Neuropsychological imaging and testing
6	Pediatric Cardiology	1) Biostatistics 2) Bioinformatics (database creation and mining) 3) Bioinformatics (next-generation sequencing analysis)^*^
7	Cardiology	1) Bioinformatics (DNA methylation analysis)
8	Pediatric Neurology	1) Bioinformatics (next-generation sequencing analysis)

* Two scholars switched mentors after 1 year when their methodologic needs changed, resulting in three RAMP-appointed mentors over the course of their KL2 awards

### Focus Group Results

In the first KL2 focus group, the scholars were asked, “Of the KL2 program elements, which did you find most helpful? Why?” Per the focus group report,

“Overwhelmingly, the scholars feel the RAMP mentorship is the most helpful program element.”

One participant was quoted as saying:

“It’s … the one resource I’ve come across that I don’t think I could replicate just by investing time … get[ing] 2 hours a month with a biostatistician, I think, without funding…. My mentors had said they don’t even have … that type of access to biostatisticians.”

The report further stated that the scholars

“unanimously feel anything involving RAMP is well worth their time” and that the scholars “believe the RAMP Mentor program should be extended to include those who don’t have a KL2 grant.”

Analysis of the second focus group found very similar themes. For example, two scholars mentioned that their RAMP Mentor “has helped a great deal with research methods.”

### KL2 Survey Results

Table 2 presents KL2 scholar self-reported experience and satisfaction with the RAMP Mentors program. All scholars “agreed” or “strongly agreed” that they were satisfied with the mentoring they received, and that it both improved the quality of their research and was complementary to the mentoring from their primary mentor. Most scholars felt comfortable approaching colleagues with methodological expertise and were confident in their ability to apply research methods discussed with their mentors. Although six of eight scholars agreed or strongly agreed that they would continue collaborating with their RAMP-appointed mentor beyond the term of the KL2 program, one reported that they would not.

**Table 2.** Self-reported KL2 scholar satisfaction with RAMP Mentors (*n* = 8)

Please indicate your level of agreement with the following statements concerning your experience in the RAMP program to date:	Strongly disagree		Disagree		Neutral		Agree		Strongly agree	
	*N*	%	*N*	%	*N*	%	*N*	%	*N*	%
My research productivity has increased because of collaborating with my RAMP Mentors	0	0	0	0	1	12.5	1	12.5	6	75
The quality of my research has improved because of collaborating with my RAMP Mentors	0	0	0	0	0	0	2	25	6	75
My comfort level approaching colleagues with methodologic expertise with research questions has increased as a result of collaborating with my RAMP Mentors	0	0	0	0	1	12.5	2	25	5	62.5
I am confident in my ability to apply the research method(s) discussed with one or both of my RAMP Mentors in collaboration with a specialist in the method(s)	0	0	0	0	1	12.5	2	25	5	62.5
The support received from my RAMP Mentors complements the support from my primary research mentor and community mentor(s)	0	0	0	0	0	0	2	25	6	75
I intend to continue collaborating with one or both of my RAMP Mentors beyond my 2-year appointment as a KL2 scholar	0	0	1	12.5	1	12.5	1	12.5	5	62.5
Overall, I am satisfied with the mentoring that I have received from my RAMP Mentors	0	0	0	0	0	0	2	25	6	75
I am satisfied with the RAMP program overall, including my interactions with program leadership and staff	0	0	0	0	0	0	2	25	6	75

Written comments on the mid-point and final surveys included:

“I expected to gain expertise in experimental design, method, and statistics. My expectations have been met and more! Probably the best part of the KL2 program in my opinion.”

“I have been super impressed with the program and it has been invaluable to me.”

“I have had substantial successes under the mentorship of my RAMP team. I was able to publish 4 manuscripts, with 2 others in preparation in just under 2 years.”

### KL2 Scholars vs. Other K Awardees at Northwestern University

**Fig. 1.**
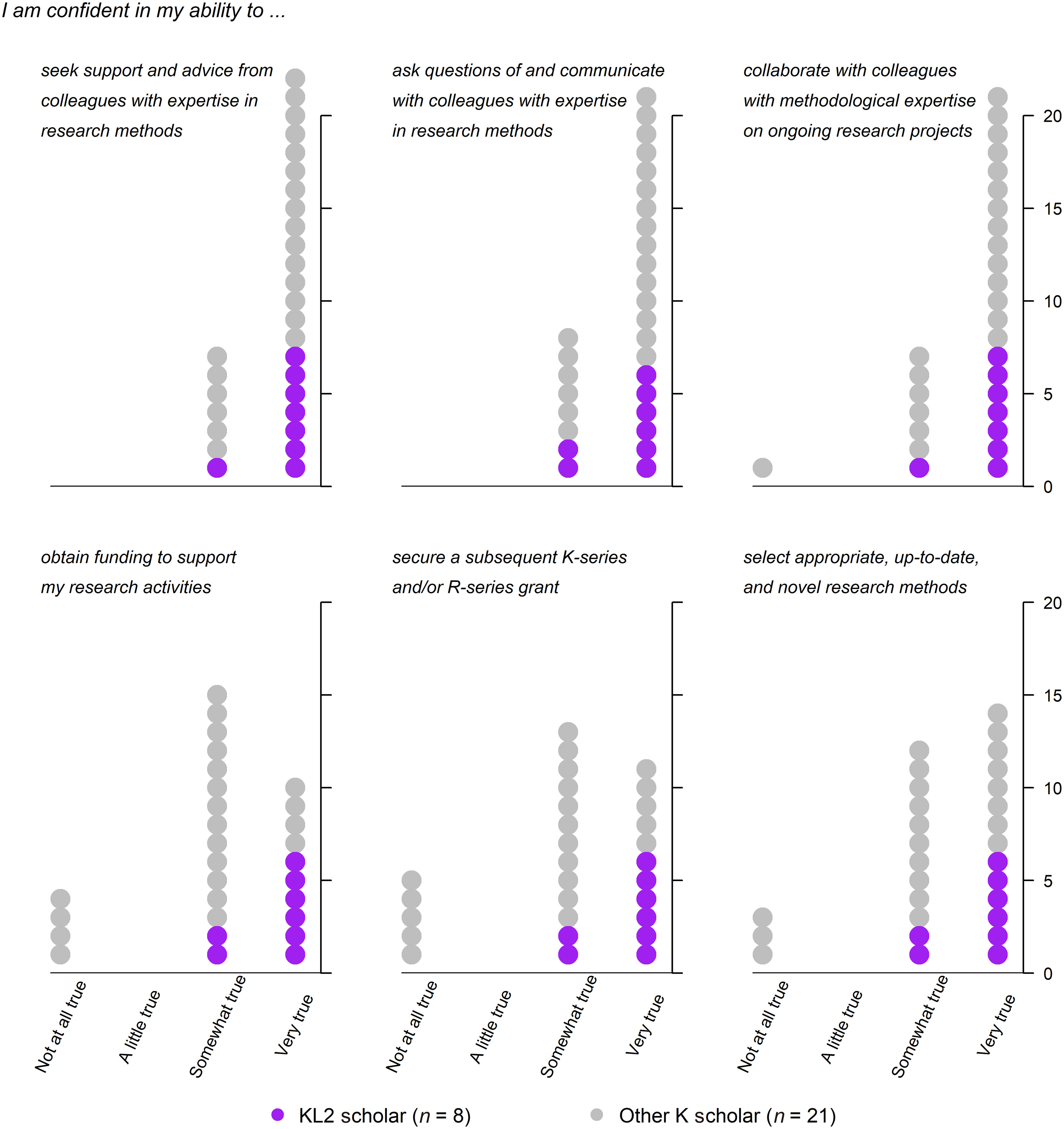
Comparison of KL2 scholar (*n* = 8) and other recent (appointed within the previous 3 years) K awardee (*n* = 21) self-reported confidence in establishing methodological collaborations and other professional milestones.

Figure 1 summarizes survey results comparing NUCATS KL2 scholars with similar Northwestern University K awardees. Results are presented for the 8 KL2 scholars and 21 respondents among the 68 Northwestern University K awardees who had been appointed within the previous 3 years of April 2018. Table 3 shows mean responses for each group of awardees, as well as the mean difference and associated 95% confidence intervals. Compared with similar Northwestern University K awardees, KL2 scholars reported greater confidence in obtaining funding to support research activities (3.8 vs. 2.8; difference 0.9, 95% CI 0.4–1.5), securing a subsequent K-series and/or R-series grant (3.8 vs. 2.8; difference 1.0, 95% CI 0.4–1.6), and selecting appropriate, up-to-date, and novel research methods (3.8 vs. 3.1; difference 0.7, 95% CI 0.1–1.2).

**Table 3.** Differences in self-reported confidence in establishing methodological collaborations and other professional milestones between KL2 scholars who participated in RAMP Mentors and other early K awardees

I am confident in my ability to …	KL2 (*n* = 8)		Other K (*n* = 21)		Mean difference	
	Mean	SE	Mean	SE	95% CI	
Seek support and advice from colleagues with expertise in research methods	3.9	0.04	3.7	0.02	0.2	−0.2, 0.5
Ask questions of and communicate with colleagues with expertise in research methods	3.8	0.06	3.7	0.02	0.0	−0.4, 0.5
Collaborate with colleagues with methodological expertise in ongoing research projects	3.9	0.04	3.6	0.04	0.3	−0.1, 0.7
Obtain funding to support my research activities	3.8	0.06	2.8	0.05	0.9	0.4, 1.5
Secure a subsequent K-series and/or R-series grant	3.8	0.06	2.8	0.05	1.0	0.4, 1.6
Select appropriate, up-to-date, and novel research methods	3.8	0.06	3.1	0.05	0.7	0.1, 1.2

### Important Modifications of RAMP to Date

From 2015 to 2019, we made minor modifications to RAMP Mentors in response to early scholar and mentor feedback. Initially, we paired each KL2 scholar with two mentors at the start of their KL2 appointment. Many scholars found it challenging to establish regular meetings with two (additional) mentors while simultaneously beginning their research project and starting associated career development activities, both individual and institutional. Beginning in September 2017, we started pairing KL2 scholars with one mentor at the beginning of their appointment. The RAMP Mentor director then checked in at 6-month intervals to determine if a second mentor was warranted. Additionally, many scholars were initially taking advantage of mentor expertise for help with current data analysis but were not fully utilizing mentors to learn about novel methods and to brainstorm future ideas. To address this, the director now distributes proposed agendas for the first five scholar/mentor meetings (see Supplementary Materials), which include the suggestion of joint discussion of a journal article highlighting a new method and a brainstorming session about the next two grants the scholar plans to submit. Finally, the program now offers scheduling support and sends calendar invitations to address the challenges busy mentors and scholars face in finding mutually agreeable times to meet.

## Discussion

In this article, we describe a novel mentorship program, RAMP Mentors, that pairs KL2 scholars with methodologic experts to promote novel, high-quality research and the development of robust research teams. Data to date are limited to eight KL2 scholars, but feedback from focus groups and surveys is extremely positive. These scholars may also have higher overall confidence in the methodologic aspects of their work, such as selecting appropriate methods or collaborating with biostatisticians, when compared with similar Northwestern University K awardees who are not part of RAMP Mentors.

There are limitations with our qualitative and quantitative assessments to date. The sample size of KL2 scholars is small and is solely determined by the number of KL2 scholar “slots” the National Institutes of Health allocates to Northwestern University within the KL2 scholars program. KL2 scholars may report satisfaction with RAMP Mentors in part because they know they are receiving extra support. Analyses comparing KL2 scholars with other Northwestern University K awardees may be biased by the modest survey response rate for other K awardees. We cannot compare the demographic characteristics between K awardees who responded and did not respond because the survey was distributed anonymously as a public link to encourage participation and honest feedback. Because RAMP Mentors is a relatively new program, we do not yet have longer-term outcomes for KL2 scholars, including additional publications or additional grant awards. We are currently collecting these data from former KL2 and other Northwestern University K awardees as it becomes available and will, in the longer term, compare these long-term outcomes. In this era of increased focus on reproducibility and rigor in research and emphasis on transdisciplinary research, we believe the mentoring and team building provided by the RAMP Mentors program will increase our scholars’ capacity to conduct high-quality research which will, in turn, increase numbers and impact of publications, rates of success at securing independent research funding, and participation in transdisciplinary projects.

We searched the academic literature to find reports of other programs focused on methodologic mentoring, particularly for investigators pursuing clinical and translational research. We found reports of two well-received, shorter-term programs: an intensive 24-day immersion program at the Clinical and Translational Science Institute at New York University [9], and a consultancy program at the University of Washington where junior faculty consulted once with a panel of experts [10]. However, we found no reports of programs similar to RAMP Mentors. As noted in the introduction, RAMP Mentors shares elements with aspects of other translational career development programs that have been highlighted as success stories or best practices. For example, the Building Interdisciplinary Research Careers in Women Health program leaders identified that one factor for success was having a team mentoring approach that included both career and content mentors [11].

From inception, a key element of RAMP Mentors has been providing salary support for mentors. The University of Utah has emphasized that a key element to their success in retention of KL2 scholars in translational science was significant support for mentoring, including up to 5% salary coverage to support mentoring efforts [12]. Typically mentors are not compensated on K awards for mentorship as the PHS 398 Training Subaward Budget Attachment specifies that salary support for mentors is not an allowable budget item (although up to $10,000 per year for associated laboratory or other research-related expenses for the mentor is permitted). However, this lack of mentor support has been identified by others as a critical issue, particularly for more junior primary investigators, with one paper stating “there is a pressing need for the research community to address the workload, institutional expectations, and reward system for research mentors” [13]. We hope that our experience can add to a productive dialog about the best ways to support research mentorship.

In response to KL2 scholar comments, we plan to expand the RAMP Mentors program to Northwestern University K awardees outside the KL2 program with our new CTSA award. We will also continue to modify the program based on scholar feedback. We will continue to collect scholar assessments of the program, with a particular emphasis on collecting metrics for biostatistical and epidemiological collaborations proposed by the BERD groups associated with the CTSA [2]. We will continue to disseminate lessons learned through the CTSA Consortium and aligned groups, such as the BERD Special Interest Group and the Association for Clinical and Translational Statisticians.

In addition to benefits to KL2 scholars, the benefits of RAMP Mentors expand to other researchers at Northwestern University. RAMP Mentors has helped methodologic faculty, many of whom are appointed on a collaborative team science faculty track (https://www.feinberg.northwestern.edu/fao/administrators/team-scientists/index.html), to partner with new collaborators. In several cases, scholar/mentor pairings have resulted in the mentor developing new research relationships with multiple investigators in the home department of the KL2 scholar. We anticipate that these new relationships will lead to additional transdisciplinary grant proposals. RAMP Mentors also raises awareness of NUCATS’ research resources, including the Biostatistics Collaboration Center, which provides biostatistics support to investigators working on grant proposals to assist with statistical analysis plans, power calculations, and other aspects of reproducibility and rigor in research proposals. We also anticipate that RAMP Mentors may serve as a model for other institutions seeking to enhance methodologic training for junior investigators, with the ultimate goal of helping them build productive multidisciplinary research teams.

## Supplementary material

For supplementary material accompanying this paper visit https://doi.org/10.1017/cts.2020.536. click here to view supplementary material
