# Supplementary material for: An innovative program to provide methodological mentoring and to foster the development of robust research teams for K awardees: RAMP Mentors
Source: J Clin Transl Sci. 2020 Sep 4;5(1):e43. doi: 10.1017/cts.2020.536 (PMC8057491; doi:10.1017/cts.2020.536)
Supplement: Supplementary file 1 [file S2059866120005361sup001.pdf]

RAMP Mentoring Program: Mentee Survey (End of Program)

The Biostatistics Collaboration Center and NUCATS Evaluation Team are surveying all RAMP mentees to assess their overall experiences with the RAMP Mentoring Program during your two-year KL2 appointment. Similar to the mid-point survey, we begin by asking you to provide feedback on your RAMP mentor(s) and then to report on your overall experience and satisfaction with the program.

Results will be reported in an aggregate form; no information that could be used to identify individual persons will be reported. Thank you for taking the time to complete this survey.

Feedback on RAMP Mentors

First, we ask you to provide feedback on your RAMP mentor(s). If you had more than two mentors over the course of your KL2 appointment, please provide feedback on the mentors you worked with most closely during the last year of the program.

Name - RAMP Mentor #1: ________________________________

Name - RAMP Mentor #2 (if applicable): ________________________________

RAMP Mentor #1

Please indicate your level of agreement with the following aspects of your interactions with your RAMP mentor during your KL2 appointment:

My RAMP mentor (#1):

	strongly disagree	disagree	neutral	agree	strongly agree	not applicable
was available to meet with me.	○	○	○	○	○	○
assisted me with implementing good research/methodological practices.	○	○	○	○	○	○
was approachable.	○	○	○	○	○	○
understood my research project and methodological needs.	○	○	○	○	○	○
gave me regular and constructive feedback on my research.	○	○	○	○	○	○
was a good fit with my own research interests and methodological needs.	○	○	○	○	○	○

advised about obtaining resources I need to accomplish my project.	◯	◯	◯	◯	◯	◯

How satisfied were you with the:

	very dissatisfied	dissatisfied	neutral	satisfied	very satisfied
frequency of meetings with this RAMP mentor?	◯	◯	◯	◯	◯
length of meetings with this RAMP mentor?	◯	◯	◯	◯	◯

RAMP Mentor #2 (if applicable)

Please indicate your level of agreement with the following aspects of your interactions with your RAMP mentor during your KL2 appointment:

My RAMP mentor (#2):

	strongly disagree	disagree	neutral	agree	strongly agree	not applicable
was available to meet with me.	◯	◯	◯	◯	◯	◯
assisted me with implementing good research/methodological practices.	◯	◯	◯	◯	◯	◯
was approachable.	◯	◯	◯	◯	◯	◯
understood my research project and methodological needs.	◯	◯	◯	◯	◯	◯
gave me regular and constructive feedback on my research.	◯	◯	◯	◯	◯	◯
was a good fit with my own research interests and methodological needs.	◯	◯	◯	◯	◯	◯
advised about obtaining resources I need to accomplish my project.	◯	◯	◯	◯	◯	◯

How satisfied were you with the:

	very dissatisfied	dissatisfied	neutral	satisfied	very satisfied
frequency of meetings with this RAMP mentor?	○	○	○	○	○
length of meetings with this RAMP mentor?	○	○	○	○	○

Overall Experience & Satisfaction with RAMP Mentors

Please indicate your level of agreement with the following statements concerning your experience in the RAMP program:

	strongly disagree	disagree	neutral	agree	strongly agree
My research productivity has increased because of collaborating with my RAMP mentors.	○	○	○	○	○
The quality of my research has improved because of collaborating with my RAMP mentors.	○	○	○	○	○
My comfort level approaching colleagues with methodologic expertise with research questions has increased as a result of collaborating with my RAMP mentors.	○	○	○	○	○
I am confident in my ability to apply the research method(s) discussed with my RAMP mentors in collaboration with a specialist in the method(s).	○	○	○	○	○
The support received from my RAMP mentors complements the support from my primary research mentor.	○	○	○	○	○
I intend to continue collaborating with any of my RAMP mentors beyond my two-year appointment as a KL2 scholar.	○	○	○	○	○

Overall, I am satisfied with the mentoring that I have received from my RAMP mentors.	○	○	○	○	○
I am satisfied with the RAMP program overall, including my interactions with program leadership and staff.	○	○	○	○	○

Please indicate how confident you feel overall in the following areas:

Today, I am confident in my ability to:

	not at all true	a little true	somewhat true	very true
seek support and advice from colleagues with expertise in research methods.	○	○	○	○
ask questions of and communicate ideas to colleagues with expertise in research methods.	○	○	○	○
collaborate with colleagues with methodologic expertise on ongoing research projects.	○	○	○	○
obtain funding to support my research activities.	○	○	○	○
secure a subsequent K-series and/or a R-series grant.	○	○	○	○
select appropriate, up-to-date, and novel research methods.	○	○	○	○

Overall, what was the most effective aspect of working with your RAMP mentors? ____________________________________

Overall, what was the least effective aspect of working with your RAMP mentors? ____________________________________

How, if at all, could the RAMP mentoring program be improved in terms of logistics, structure, etc. to better meet your needs and/or the needs of future mentees? ____________________________________

What, if any, "pain points" did you experience with the RAMP mentoring program, either associated with an aspect of the program or with a specific mentor? ____________________________________

How would you describe the RAMP mentoring program to a future K scholar? What could they expect to gain by participating? What would they need to know or do to get the most out of the experience? ____________________________________

Research Methods K Survey

Please complete the survey below.

Thank you!

I am confident in my ability to: (Please indicate how confident you feel at this time in the following areas on a four-point scale from "not at all true " to "very true.")

	not at all true	a little true	somewhat true	very true
1) seek support and advice from colleagues with expertise in research methods.	○	○	○	○
2) ask questions of and communicate ideas to colleagues with expertise in research methods.	○	○	○	○
3) collaborate with colleagues with methodological expertise on ongoing research projects.	○	○	○	○
4) obtain funding to support my research activities.	○	○	○	○
5) secure a subsequent K-series and/or an R-series grant.	○	○	○	○
6) select appropriate, up-to-date, and novel research methods.	○	○	○	○

7) At this time I am supported on the following type of career development award (select one):

- ◯ PPSRA
- ◯ K01
- ◯ K02
- ◯ K07
- ◯ K08
- ◯ K12
- ◯ K23
- ◯ K25
- ◯ K99
- ◯ other

K award if other: ________________________________

8) I've had the support of my current K award for (select one):

- ◯ < 1 year
- ◯ 1 - 2 years
- ◯ 3 - 4 years
- ◯ > 4 years
- ◯ 4+ years

9) At a previous time I was supported on the following type of career development award(s):

- ☐ None, this is my first career development award.
- ☐ PPSRA
- ☐ K01
- ☐ K02
- ☐ K07
- ☐ K08
- ☐ K12
- ☐ K23
- ☐ K25
- ☐ K99
- ☐ other

Previous K award if other: ________________________________

Any additional information: ____

RAMP Mentors Monthly Meetings

KL2 Scholars and their RAMP Mentors should meet at least monthly; suggestions for the first five meetings are outlined below. *The goal of the meetings are to provide the KL2 Scholar with the resources and tools to conduct and oversee primary data analysis on their own.* We recommend that meeting agenda be shared by the Scholar and RAMP Mentor at least 1 week in advance of meeting. A 2-3 sentence summary of each meeting should be logged. Eventually, we recommend that the scholar and the mentor alternate setting meeting agendas. In some cases it may be advantageous for the mentee to meet with both RAMP mentors simultaneously (when applicable), although this is not required.

Meeting #1: Introductions

Have RAMP Mentor read the KL2 application, then Scholar will walk the Mentor through the project. RAMP Mentor also explains his or her area of expertise and how that area fits in to the project. We recommend you consider including your KL2 mentors in this first meeting.

Meeting #2: Methodologic considerations

Together outline all methods aspects of the project, including analysis pipelines, data capture, data management, and analysis (*this includes areas that may not fall within the RAMP mentors areas of expertise).* Identify which aspects each RAMP Mentor will be directly involved in (and note if there are areas that might benefit from a second or different RAMP mentor in the future). Develop timelines for methods-related items, e.g. when data capture will be complete, when statistical analyses should begin and when you anticipate analyses to be completed.

Meeting #3: Topics to be covered in future meetings

Generate a list of topics/questions to focus on during upcoming meetings that will help prepare the Scholar both for the specific project and for generally conducting research. Examples of topics are how to conduct reproducible research, generating effective figures and tables, aspects of study design (e.g. case-control versus cohort study), or the necessarily elements required for the calculation of a power calculation.

Meeting #4: Specific topic from previous meeting

Cover one of the most important topics brainstormed at the last meeting.

Meeting #5: Review work in progress

The Scholar should come with a product (abstract, poster, manuscript) that is in progress for review.

Additional Considerations:

We recommend that a recurring agenda item be any new analytic or methodologic issues that have arisen in publications or grant proposals in progress. Additional topics of discussion may include (but are not limited to):

- Careful review of one or two recent publications or presentations in a relevant field
- Discussion of a newly developed methodology
- Guided tutorial of relevant analytic software
- Brainstorming of future grant ideas
